# Ship wave patterns on floating ice sheets

**DOI:** 10.1038/s41598-022-23401-8

**Published:** 2022-11-07

**Authors:** Kristoffer Johnsen, Henrik Kalisch, Emilian I. Părău

**Affiliations:** 1 Department of Mathematics, University of Bergen, Postbox 7800, 5020 Bergen, Norway; 2 School of Mathematics, University of East Anglia, Norwich Research Park, Norwich, NR4 7TJ UK

**Keywords:** Fluid dynamics, Applied mathematics, Natural hazards

## Abstract

This paper aims to explore the response of a floating icesheet to a load moving in a curved path. We investigate the effect of turning on the wave patterns and strain distribution, and explore scenarios where turning increases the wave amplitude and strain in the ice, possibly leading to crack formation, fracturing and eventual ice failure. The mathematical model used here is the linearized system of differential equations introduced in Dinvay et al. (J. Fluid Mech. 876:122–149, 2019). The equations are solved using the Fourier transform in space, and the Laplace transform in time. The model is tested against existing results for comparison, and several cases of load trajectories involving turning and decelerating are tested.

## Introduction

Hydro-elastic waves on frozen lakes and sounds can by excited by moving loads such as motorized vehicles. Observations of such waves using satellite synthetic-aperture radar (SAR) imagery reveal ship-wave like patterns which compare favorably with results from earlier theoretical work^1–3^.

The study of hydro-elastic waves has a long history going back to the 1950’s and was prompted by attempts to systematically use solid ice covers as a means of mechanized transportation. In cold regions today, some winter truck routes are partially over ice-covered lakes, as this routing provides a low-cost alternative to building asphalt roads running along the lakeshores^4^. In some cases, these ice roads are the only economical means of transportation to reach remote communities in the North. These routes are also of major importance for mining operations in several northern locations which depend on high-volume low-cost shipping of tools, equipment and lore.

Authorities in the northern regions follow various plans for opening and closing ice roads, maintaining safety by checking ice thickness, temperature and consistency, weather conditions, and planning routes based on local conditions and operational experience. Maximal permitted loading is in many cases based on Gold’s formula which relates the thickness of the ice cover to the allowable load based largely on empirical observations of ice failure or non-failure under various loading conditions^5^.

In the case of heavy moving loads, the speed of the load is also an important factor in maintaining safety of ice roads. Indeed, it is well known that large speeds can create resonant waves in the ice cover, and under certain conditions of speed, ice thickness, and water depth, the deflection under a vehicle traveling on a floating ice sheet may be amplified considerably. Under operational conditions, a speed limit of 15 mph (24 km/h) is often imposed, and an important component of ice road safety is proper instruction of truck operators.

Exceeding the speed limit may lead to crack formation, and especially near the shore to so-called blowouts. Blowouts are usually caused by pressure buildup due to constructive interference of waves excited by heavy moving vehicles during shore approaches. Once a blow-out hole has formed, subsequent traffic must be rerouted.

While many early studies involving moving loads relied on constant load speed^3^, the importance of incorporating transients was already highlighted in^6,7^. Non-constant load speeds are included in a few studies^8–12^, and in particular it was shown that a decelerating load could lead to constructive interference of waves which could exceed the critical stress and thereby lead to crack formation^8,12^. While some studies examine non-homogeneous ice conditions^13,14^ and damping properties of the ice cover^15–20^ which can be a major factor in ice failure, the present study is focused on the effect of changes in speed and in particular changes in the direction of propagation of the moving load.

**Figure 1:**
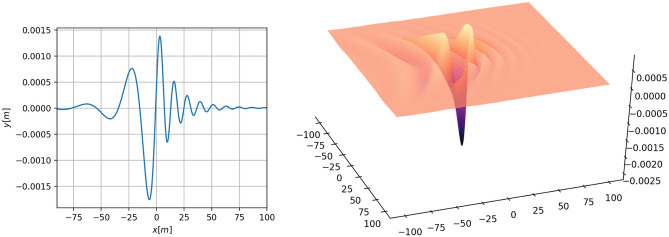
Hydro-elastic wave response to a load traveling at a velocity $v=7$ m/s. The critical speed in this situation is $v_c=5.94$ m/s. The left panel shows the central line of the graph in the right panel. At the time shown in this figure, the load is positioned at $x = 0$. The depth is $H=6.8$ m, thickness of the ice sheet is $h=0.17$m, and the Young modulus is $E=5.1\times 10^8$N/m^2^. The fluid density is 1026 kg/m^3^, and the ice density is 917 kg/m^3^. The damping coefficient is $b=788$kg m$^2$/s^8^. The mass of the load is 235 kg. Axis units are in meters.

In the existing literature on theoretical modeling of hydroelastic waves induced by a moving load the focus has been exclusively on loads moving in a *straight path*, such as shown in Fig. 1. In the present contribution, we investigate the effect of a *curved path* on the waves created by the moving load. As already intimated above, curved vehicle trajectories are of interest because turning may sometimes be unavoidable due to routing problems, obstacles on the ice or localized ice failures. As will be shown in the body of this article, turning may also lead to constructive interference which may be more dangerous than slowing down.

## Mathematical model for hydro-elastic waves

We consider an inviscid, incompressible fluid of density $\rho$ and undisturbed depth *H*, covered by a thin elastic solid of density $\rho _I$ described by the Kirchhoff–Love plate theory^21^. For the purpose of describing hydro-elastic waves, one may assume irrotational flow, so that the flow in the fluid foundation is described by a potential $\phi (x,y,z,t)$ satisfying Laplace’s equation $\bigtriangleup \phi = 0$. We assume that the thickness *h* of the elastic solid is small compared to both the depth and a typical wavelength. This simplification allows us to use a common coordinate system for the fluid and solid, and we specify that the fluid-solid interface is located at $z=\eta (x,y,t)$. At the interface, the trace of the velocity potential is defined by $\Phi (x,y,t) = \phi (x,y,\eta (x,y,t),t)$.

Using Hooke’s law together with the second Kirchhoff hypothesis (i.e. assuming that deformations are entirely due to bending, and disregarding transverse deformations), the boundary condition at the solid-fluid interface can be written in terms of the hydroelastic parameter $\kappa$ as a viscoelastic Bernoulli equation in the form

1 $$\begin{aligned} \kappa g \triangle _H^{2} \eta - \frac{\rho _I h^3}{12 \rho } \partial _t^2 \triangle _H \eta + \frac{\rho _I h}{\rho } \partial _t^2 \eta + \frac{b}{\rho } \partial _t \eta + g \eta + \phi _t + \frac{1}{2} | \nabla \phi |^2 + \frac{P}{\rho } = 0. \end{aligned}$$

In this equation, $\bigtriangleup _H = \partial _x^2 + \partial _y^2$ is the two-dimensional Laplacian. The parameter $\kappa$ is defined in terms of the flexural rigidity ${\mathscr {D}}$, the gravitational acceleration *g* and the fluid density $\rho$ by $\kappa = \frac{{\mathscr {D}}}{\rho g}$. The flexural rigidity measures the resistance of the ice cover to bending, and is usually given in terms of the ice thickness *h*, the Young modulus *E* and the Poisson ratio $\sigma$ as ${\mathscr {D}} = \frac{E h^3}{12(1-\sigma ^2)}$. The parameter *b* in (1) is a damping coefficient and *P*(*x*, *y*, *t*) denotes the pressure imposed by the load.

The second term in Eq. (1) takes account of horizontal acceleration in the solid. This effect is often neglected in the study of hydro-elastic waves, but in the present work this term is kept in the equation as it allows improved handling of the pressure forcing.

In virtually all cases where the ice can safely support a load, the deflection of the ice sheet is on the order of a few centimeters which is small compared to all other length scales in the problem. As a result, it is generally a good approximation to consider the linear wave dynamics given by the linearized form of the equations. A particularly useful version of the equations is written in terms of the so-called Dirichlet Neumann operator $G_0$ relating Dirichlet to Neumann boundary data for the potential in the fluid foundation^22,23^. In terms of the interface deflection $\eta (x,y,t)$ and the trace of the potential $\Phi (x,y,t)$, the equations are written in the form

2 $$\begin{aligned} \eta _{t}&= G_0 \Phi , \end{aligned}$$

3 $$\begin{aligned} \Phi _{t}&= - g \dfrac{1 + \kappa \triangle _H^{2}}{K}\eta - \dfrac{b}{\rho }\dfrac{G_{0}}{K}\Phi - w, \end{aligned}$$

with the pressure forcing given in terms of $w = \frac{K^{-1}}{\rho } P(x,y,t)$. The equations are derived in^8^ based on the approach used in^24,25^. The first equation is a linearized version of the kinematic boundary condition. The operator 1/*K* is defined as the inverse of

4 $$\begin{aligned} K = 1 + \dfrac{\rho _{I}h}{\rho }\left( 1-\dfrac{h^{2}\triangle _H}{12}\right) G_{0}. \end{aligned}$$

It is shown in the Appendix, how the operators *K* and $G_0$ can be written more explicitly using the two-dimensional Fourier transform. The system of Eqs. (2)–(3) accurately describes the dynamics of a wave of arbitrary wavelength, and is therefore known as a fully dispersive system^24,26^.

The system can be solved using the Laplace transform ${\mathscr {L}}$. Indeed, defining the operators

$$\begin{aligned} R = \dfrac{bG_{0}}{2 \rho K} \ \ \text{ and } \ \ U = \sqrt{\dfrac{g(1+\chi \triangle _H^{2})G_{0}}{K} -R^{2}}, \end{aligned}$$

then for zero initial data, the solution takes the form $\eta = {\mathscr {L}}^{-1}({\hat{m}}(s)\cdot {\hat{w}}(s))$, where ${\hat{m}}$ denotes the Laplace transform of $m ={\mathscr {L}}^{-1}((\frac{-G_{0}}{(s+R)^{2} + U^{2}})$ and ${\hat{w}}$ denotes the Laplace transform of $w = \frac{K^{-1}}{\rho }\cdot P(x,y,t)$.

As is customary, an expression for $\eta (\cdot ,t)$, can be found using the convolution identity

5 $$\begin{aligned} {\mathscr {L}}^{-1}({\hat{m}}(s)\cdot {\hat{w}}(s)) = {\mathscr {L}}^{-1}({\hat{m}}(s))*{\mathscr {L}}^{-1}({\hat{w}}(s)) = m * w, \end{aligned}$$

defined explicitly as

$$\begin{aligned} \eta (\cdot ,t) = {\mathscr {F}}^{-1} \int _{0}^{t}m(t-\tau ) {\mathscr {F}}w (\tau ) d\tau , \end{aligned}$$

where ${\mathscr {F}}$ and ${\mathscr {F}}^{-1}$ denote the Fouier transform and inverse Fourier transform, respectively. The integral kernel *m* is given by the inverse Laplace transform ${\mathscr {L}}^{-1}({\hat{m}}(s))$ which can be solved exactly and is written in the form

6 $$\begin{aligned} {\mathscr {L}}^{-1}({\hat{m}}(s))(t) =\dfrac{G_{0}}{2 i U}\left( e^{-t(R-iU)}-e^{-t(R+iU)}\right) . \end{aligned}$$

In some works, the load is assumed to be rectangular, but due to the inclusion of rotary inertia and the scale separation between the load and the wavelength of the excited waves, one my also consider a point load. In the present case, since the load is following a curved path, it is most expedient to use either a point load, or a symmetric Gaussian distribution which will be rotation-invariant under the change in orientation which occurs while turning. Assuming for the moment that we are dealing with a point load with mass $w_0$, which is following a path parametrized by the vector $\vec {X}(t) = \left[ x(t),y(t) \right]$, the time evolution of the load position is given in terms of the Fourier transform as $w(\cdot ,t) = w_0 {\mathscr {F}}^{-1} e^{i\vec {X}(t) \cdot \vec {\xi }}$. It then transpires that the deflection has the form

7 $$\begin{aligned} \int _{0}^{t}m(t-\tau ) w_{0} e^{i\vec {X}(t)\cdot \vec {\xi }}d\tau , \end{aligned}$$

where $\vec {\xi } = (\xi _{1},\xi _{2})$ is a vector in Fourier or wavenumber space. Since the Fourier multiplier operator *m*(*t*) is given in explicit form, the general solution can be written as

8 $$\begin{aligned} \int _{0}^{t}\dfrac{G_{0} w_0}{2 i U}\left( e^{-(t-\tau )(R-iU)}-e^{-(t-\tau )(R+iU)}\right) e^{i \vec {X}(\tau )\cdot \vec {\xi }}d\tau . \end{aligned}$$

For reasons that will be apparent later it is convenient to keep the solution as two separate integrals, but it can also be written in the tidy form

9 $$\begin{aligned} \eta (\cdot ,t) = {\mathscr {F}}^{-1} \dfrac{G_{0} w_0}{U}\int _{0}^{t}e^{-(t-\tau )R} e^{-i\vec {X}(\tau )\cdot \vec {\xi }} \sin \left[ (\tau -t)U\right] d\tau . \end{aligned}$$

This integral can be solved analytically in some cases, but in general it has to be evaluated numerically. In the case of a Gaussian load distribution, the integrand will contain the term $e^{i \vec {X}(\tau )\cdot \xi } {\mathscr {F}}w_0$, but the final solution may also be written in explicit form.

Given a solution $\eta (\cdot ,t)$, the corresponding shear strain can be computed using linear strain theory as used for example in^27^. In order to obtain the maximum strain, one may use the maximum eigenvalue of the Hessian matrix $\sigma _{ij} = \partial _{i}\partial _{j}\eta \cdot \dfrac{h}{2}$ scaled by *h*/2 where we recall that *h* is the thickness of the ice sheet. Since the evaluation of the strain involves second derivatives, a point load will lead to singularities, so that it is best in this case to use a Gaussian weight distribution.

## Numerical methods

In order to compute the deflection of the ice sheet due to a moving load, the various integrals given above need to be evaluated. In addition, since the spatial operators are given in terms of Fourier multipliers, the FFT and inverse FFT are used. For this purpose, we define a discrete wavenumber vector $\vec {k}(n) = [k_1(n),k_2(n)]$ , where $n = [-N/2-1,N/2]$.

While it is possible to use a point load without any problem, the computations are more stable (in particular when approximating the strain) so all results here are given for a load with a circular Gaussian weight distribution.

**Figure 2:**
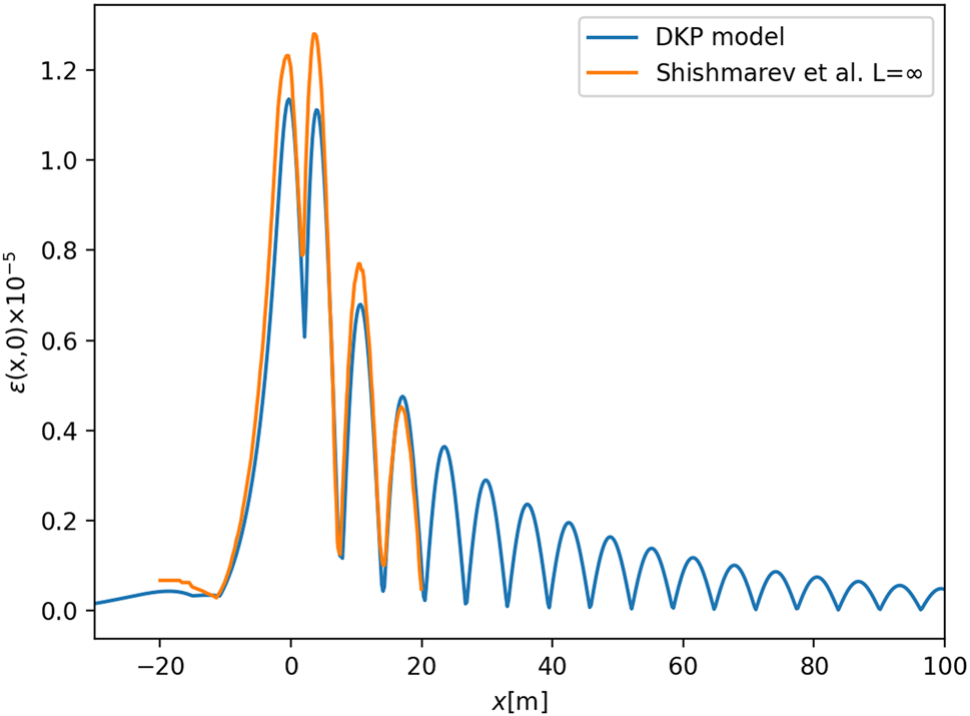
Strain in ice due to wave excited by moving load. In this case, the depth is $H=2$m, the ice parameters are $h=0.1$m, $E=4.2\times 10^9$N/m^2^, and otherwise as in Fig. 1. The load is 100 kg, moving at a speed of 25 km/h. These values are the same is in^27^.

The integration procedure is explained by looking at an example of a path with two parts. Consider a straight path parametrized by $X(t) = X_1(t), \text{ for } 0 \le t < c$ is followed by a turn parameterized by $X_2(t), \text{ for } c< t < T$. In this case, then it is convenient to split the integral for the solution (8) into two parts. If we want a part of the first solution we only need to integrate over $X_1$ but if we afterwards want the solution on the second part of the path, we still have to evaluate the whole integral

10 $$\begin{aligned} \dfrac{G_{0} w_0}{2 i U} \left( \int _{0}^{t}(e^{-(t-\tau )(R-iU)}e^{i\vec {X}(\tau )\cdot \vec {\xi }}d\tau - \int _{0}^{t}e^{-(t-\tau )(R+iU)}e^{i\vec {X}(\tau )\cdot \vec {\xi }}d\tau \right) . \end{aligned}$$

If we define the integrals over $X_1(t)$ as

11 $$\begin{aligned} I_1 = \int _{0}^{c}(e^{-(t-\tau )(R-iU)}e^{i\vec {X}(\tau )\cdot \vec {\xi }}d\tau , \; \; I_2 = \int _{0}^{c}(e^{-(t-\tau )(R+iU)}e^{i\vec {X}(\tau )\cdot \vec {\xi }}d\tau , \end{aligned}$$

then the final solution for $t>c$ can be written as

12 $$\begin{aligned} \dfrac{G_{0} w_0}{2 i U} \left( e^{-(t-c)(R -iU)} \,I_1 + \int _{c}^{t} e^{-(t-\tau )(R-iU) - i\vec {X}(\tau )\cdot \vec {\xi }} d\tau - e^{-(t-c)(R + iU)} \, I_2 - \int _{c}^{t}e^{-(t-\tau )(R+iU) -i\vec {X}(\tau )\cdot \vec {\xi }} \right) . \end{aligned}$$

**Figure 3:**
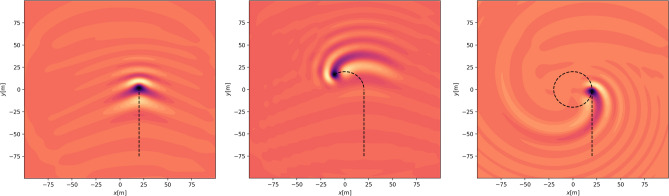
Wave pattern excited by a vehicle of mass 235 kg on an ice plate of thickness $h=0.17$m over a fluid foundation of depth $H=6.8$m. Left panel shows the vehicle at the beginning before the turn has been started ($t=0.32s$). Center panel shows the wave pattern while the vehicle is turning at $t=6.5$s. Right panel shows the wave pattern after one full turn has been completed at $t=19$s.

**Figure 4:**
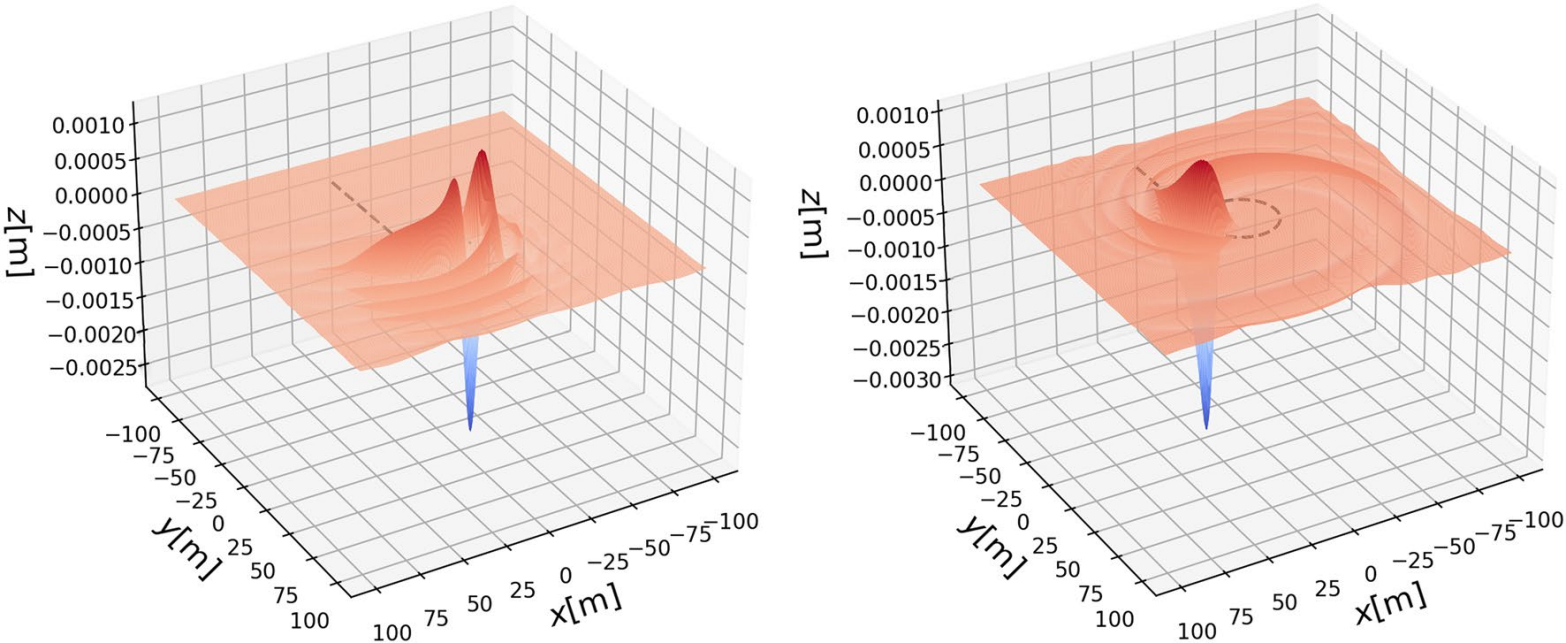
Left panel: Three-dimensional wave pattern at $t=6.5$s. Right panel: Three-dimensional wave pattern at $t=19$s. The parameters are the same as in Fig. 3.

**Figure 5:**
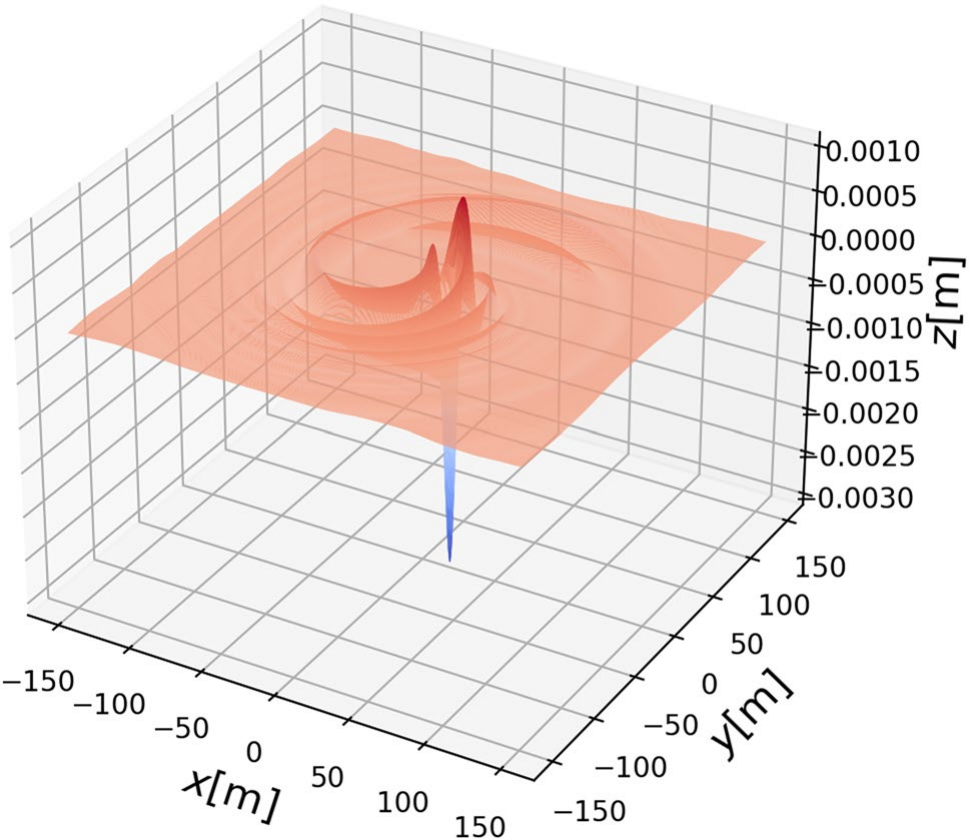
A three-dimensional plot of the wave response at $t = 38.7$s. Note that a quasi-steady wave pattern emerges as the vehicle continues to go in a circle. The parameters are the same as in Fig. 3.

Of course, depending on the situation, one may split the integral at any point along the path, and one could even define the solution at any time using an iterative process on discrete time steps. Another way to proceed is to also compute $\Phi (x,y,t)$, use the previous time step as initial condition, and propagate the solution that way. An example of the integral method is shown in the contour plots in the next section (Figs. 3, 4 and 5), where a straight path is followed by a circular path.

First, in order to test our solution strategy and the numerical implementation, we compare the strain computed based on our solutions with the results obtained in^27^. In that work, the ice cover is fixed to vertical walls at the boundary, so that some deviation is to be expected. Nevertheless, comparing the results from the present study to the strain computed in^27^, one may conclude that the results line up quite well, at least for the central line $\epsilon (x,0)$ shown in Fig. 2.

## Results

### Ship-wave patterns for circular paths

In a situation with a given set of parameters, in particular ice thickness *h*, fluid depth *H*, and flexural rigidity ${\mathscr {D}}$ there is a critical velocity $v_c$. A load moving at a speed below $v_c$ cannot excite resonant waves in the hydro-elastic system, and this velocity range is known as the quasi-steady regime. In the following, we illustrate the response to various paths taken by a load moving at a super-critical speed. In the examples shown below, we are looking at the case when the depth of the fluid base is $H = 6.8$m the thickness of the ice sheet is $h = 0.17$m, and the flexural rigidity is ${\mathscr {D}} = 2.35\times 10^5$Nm, so that the the hydro-elastic parameter is $\kappa = 23.3$m$^4$, and the critical load speed is $v_c = 6.0$m/s. (similar to the field parameters found in^28,29^).

First, we display the “ship wave pattern” excited by a load moving in a straight path (Fig. 3, left panel). Then we display how the pattern changes when part of the path is curved (Fig. 3, center panel). Finally in the right panel of Fig. 3, we show the pattern excited by a load which moves in a straight path followed by a full circle. Due to damping, the waves excited during the straight part of the path are already too small to be visible in the right panel of Fig. 3. Figure 4 shows a three-dimensional plot of the wave response to a moving load in a partial or full circle, corresponding to the center and right panels of Fig. 3.

Finally, Fig. 5 shows the wave pattern after the vehicle continues moving in the same circle. In this case, due to the damping, a quasi-steady wave pattern emerges which appears to move outward in a spiral pattern. This type of behaviour may grant further investigation as various interesting wave patterns may emerge in this situation. However in the present work, we are interested in safety aspects of ice roads, and we now turn to the potential danger incumbent in making turns of varying radius.

### Turning on an ice road

As mentioned in the introduction, ice roads are a vital part of the infrastructure in cold regions such as Canada, supporting remote communities and mining operations^4^. Popularized by epic TV productions such as *Ice Road Truckers*, ice roads have also become somewhat of a tourist attraction^30^. In the age of global warming, ice thickness variations have become more unpredictable, and the managing and maintenance of ice roads has become more difficult^4^. Maximal loading is generally based on Gold’s formula, usually given in the form ${\mathscr {P}} = {\mathscr {A}} h^2$, where *h* is the ice thickness, ${\mathscr {P}}$ is the maximal mass, and ${\mathscr {A}}$ is a constant. Values between ${\mathscr {A}}=3.5$kg/cm$^2$ and ${\mathscr {A}}=7$kg/cm^2^ are usually required for normal operations, but different authorities use various values for road determining road closures. In each case, the level of acceptable risk must be weighed against demand, and further operational controls should be implemented if higher values of ${\mathscr {A}}$ are to be used.

If many inexperienced drivers such as tourists use the ice roads, it can have an impact on the overall safety of the roads. All ice roads require strict speed limits which range from 4 mph (6.5 km/h) on very shallow lakes to 15 mph (24 km/h) on most roads and up to 22 mph (35 km/h) on deep lakes^31^. It is well known that if a vehicle exceeds the critical speed for a particular configuration depending on the depth of the fluid foundation, the ice thickness and the ice consistency (e.g. salinity, temperature, enclosed impurities), resonances in the hydro-elastic system create waves propagating independently of the load. Especially in near-shore locations, these waves can create dangerous ice excursions as the waves may interact with the shoreline, and the reflections can interfere constructively with the still incoming waves. This may lead to so-called blowouts, often in the near-shore region. Due to blowouts and other obstacles, rerouting of traffic may become necessary. In what follows, we examine the safety of turning on the ice.

We consider an idealized case where a vehicle travels at a relatively high speed of 22 mph (35 km/h) a speed that might be considered safe on a deep lake. If the vehicle enters a shallower area either nearshore or due to a shoal in the lake, it might be in a position where the critical speed is much lower.

If there is a blow-out region or an obstacle ahead, the vehicle will either have to slow down or turn in order to avoid the obstacle. Imagine that at 8.9 mph (14.4 km/h) the driver contemplates turning. Because of the decreasing depth, the critical speed is now only 7 mph (11.3 km/h), so the vehicle travels at supercritical speed. For the sake of being explicit, we look at the case of a 1000 kg vehicle on an ice cover of thickness $h=0.2$m. This would correspond to using Gold’s formula with a rather conservative choice of the constant ${\mathscr {A}}=2.5$kg/cm^2^.

**Figure 6:**
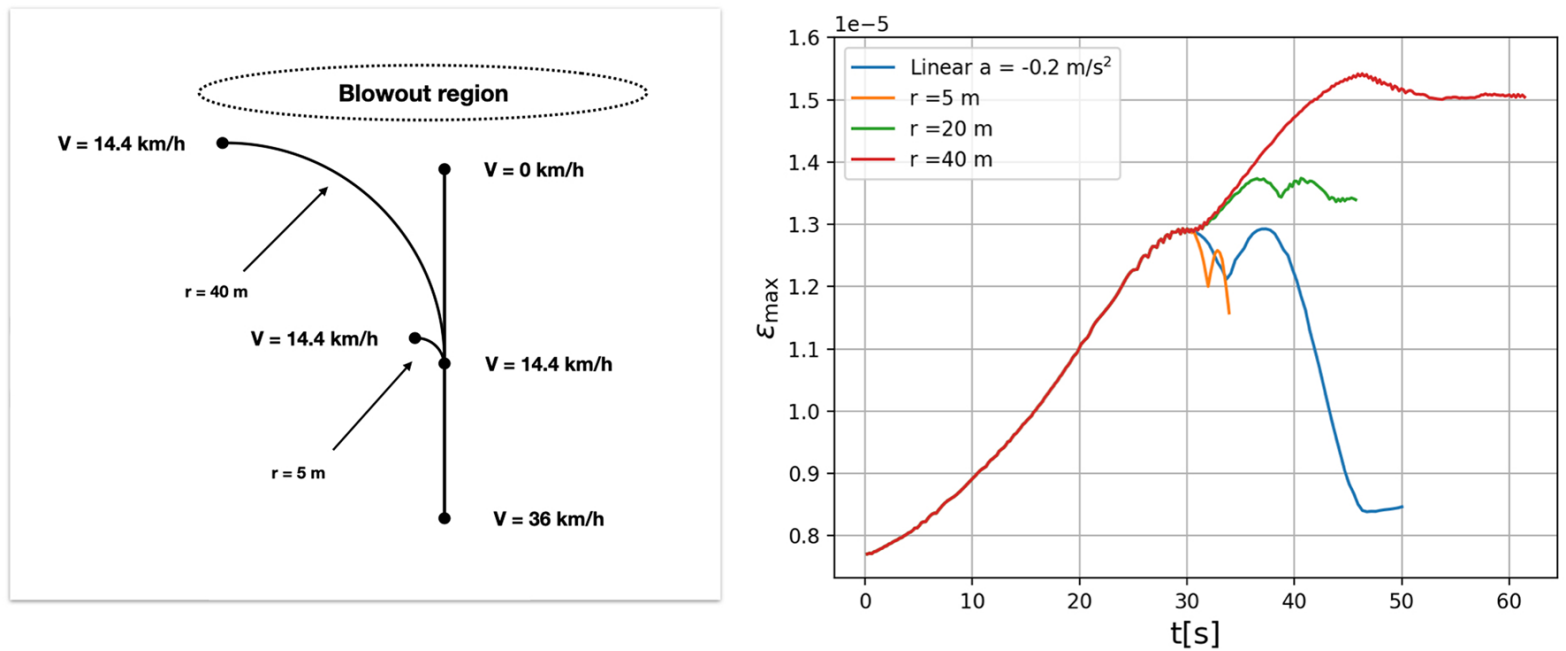
Navigating a blowout. Left panel: Different routes of a vehicle aiming to avoid an obstruction in the ice. The vechicle first slows from 34 to 14 km/h on a linear path. Then there are three options: a path with radius 5 m, a path with radius 40m, or a continued linear path and continued deceleration at a constant rate of $a=-0.2$ m/s. Right panel: Maximum strain as a function of time for a 1000 kg vehicle continuing in a straight path and slowing down, or tracing out curves at various angles without slowing down further.

From the plots shown in Fig. 6 and the numbers given in Table 1, the most dangerous action seems to be turning with a large radius (red and green curves in Fig. 6). This path puts a lot more stress on the ice over and for a greater time. For the 5-m turning radius, the strain in the model actually decreases, but as traction will always be a problem when driving on ice a turn with a 5-m radius might be unfeasible. Therefore, even if aiming for a sharp turn, the driver might end up in a turn with a larger radius adding a potentially uncontrolled vehicle to the mix. The safest behaviour in this case would to try to stop in front of the blow-out, though this may not be possible due to reduced tire traction on ice.

**Table 1:** For the vehicle going into a turn, the maximal strain was computed for various vales of the radius.

Turning radius (m)	30	50	60	70	80	90	Inf
Maximum strain	1.468−05	1.581−05	1.599−05	1.608−05	1.594−05	1.575−05	1.457−05

The parameters are the same as in Fig. 6. The maximume strain occurs at a radius of $\sim 70$m. The strain then appears to decrease with increasing radius, until it eventually converges to the value for a straight vehicular path.

## Conclusions

The subject of this paper has been the description of wave patterns induced by loads moving on ice sheets in curved paths. It was shown how the model (2)–(3) can be solved explicitly for a load traveling in a curved path. While the present work concentrated on point loads and Gaussian distributed loads, any other footprint and weight distribution of load can be handled by our approach. Loads moving in a straight path and in a circular path have been considered, but the method laid out here applies to an arbitrary curved load path.

It is well known that on ice roads, speed related blowouts may occur. Ice roads are particularly treacherous near the shore, as the critical speed is smaller due to smaller depth, and wave reflecting off the shore may combine with waves generated by a moving vehicle to crack the ice. Even for expert operators, keeping below the critical speed may be difficult at times, as the most important factor is the depth of the water beneath the ice. If conditions are right the critical speed can drop as low as 10 km/h^4^, and slowing down followed by turning may in some cases be dangerous even in conditions which would be otherwise considered safe. Indeed it has been shown that changes in direction can have a significant effect on the strain in the ice induced by the waves excited by the moving load.

## Figures and Tables

**Figure 7:**
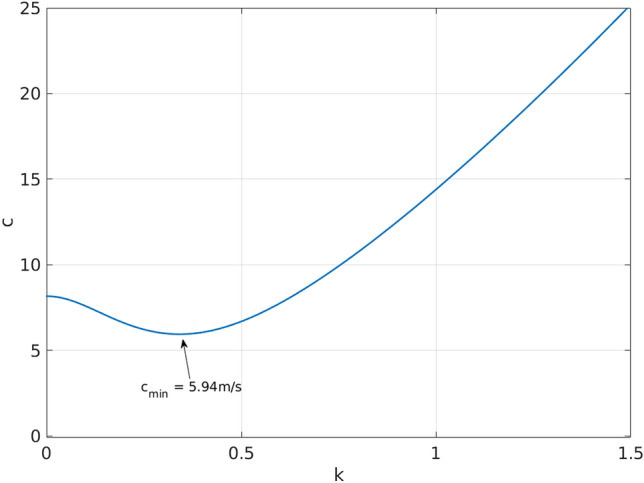
The phase speed *c* of a free wave plotted against the wavenumber *k* according to the dispersion relation (22). The parameters are the same as in Fig. 1: the depth is $H=6.8$m, the thickness of the ice sheet is $h=0.17$m, the Young modulus is $E=5.1\times 10^8$N/m$^2$, the fluid density is 1026 kg/m$^3$, and the ice density is 917 kg/m$^3$. Axis units are [*m*/*s*] for vertical axis and [1/*m*] for horizontal axis.

## Data Availability

The datasets generated and analyzed in the current study are available from the corresponding author on reasonable request. The *python* code used to generate the data can be found at Github: https://github.com/krizz227/IceWaveTools.

## Appendix

### Fourier multiplier operators

For the convenience of the reader, we recall the definition of various Fourier multiplier operators. The operators $G_0$ and *K* are defined in terms of the two-dimensional Laplacian $\bigtriangleup _H = \partial _x^2 + \partial _y^2$ as

13 $$\begin{aligned} G_{0} = \sqrt{\triangle _H} \tanh (H\sqrt{\triangle _H}), \end{aligned}$$

$$\begin{aligned} K = 1 + \dfrac{\rho _{I}h}{\rho }\left( 1-\dfrac{h^{2}\triangle _H}{12}\right) G_{0}. \end{aligned}$$

These operators can be written in terms of the Fourier transform

14a $$\begin{aligned} {\hat{f}}(\xi _1,\xi _2)={\mathscr {F}}\{f(x,y)\}= \int _{-\infty }^\infty f(x,y) e^{-i\xi _1x-i\xi _2y} \, \mathrm {d}x\mathrm {d}y, \end{aligned}$$

and the inverse Fourier transform

14b $$\begin{aligned} f(x,y)={\mathscr {F}}^{-1}\{{\hat{f}}(\xi _1,\xi _2)\} = \frac{1}{2\pi }\int _{-\infty }^\infty {\hat{f}}(\xi _1,\xi _2) e^{i\xi _1x+i\xi _2y} \, \mathrm {d}\xi _1 \mathrm {d}\xi _2. \end{aligned}$$

We have

15 $$\begin{aligned} G_{0} = {\mathscr {F}}^{-1} \Big \{ {\scriptstyle \sqrt{\xi _{1}^{2}+\xi _{2}^{2}} } \, \tanh \big ( H {\scriptstyle \sqrt{\xi _{1}^{2}+\xi _{2}^{2}}} \big ) \Big \}, \end{aligned}$$

so that $$G_0 f = {\mathscr {F}}^{-1} \Big \{ {\scriptstyle \sqrt{\xi _{1}^{2}+\xi _{2}^{2}} } \, \tanh \big ( H {\scriptstyle \sqrt{\xi _{1}^{2}+\xi _{2}^{2}}} \big ) \Big \} {\mathscr {F}} f.$$

Similarly, we have

16 $$\begin{aligned} K = {\mathscr {F}}^{-1} \Big \{ 1 + \dfrac{\rho _{I}h}{\rho }(1+\dfrac{h^{2}}{12}(\xi _{1}^{2}+\xi _{2}^{2}))\sqrt{(\xi _{1}^{2}+\xi _{2}^{2})}*\tanh \left( H \sqrt{(\xi _{1}^{2}+\xi _{2}^{2})}\right) \Big \}. \end{aligned}$$

### Derivation of the linearized system

A derivation of the linear system (2)–(3) is now given. Since the fluid base is incompressible, and assuming that the flow is irrotational, the governing equations consist of the Laplace equation

17 $$\begin{aligned} \phi _{xx} + \phi _{yy} + \phi _{zz} = 0 \quad \text{ for } \quad x,y \in {\mathbb {R}}, \quad -H< z < 0 , \end{aligned}$$

The Neumann boundary condition at the flat bottom

18 $$\begin{aligned} \phi _z = 0 \quad \text{ at } \quad z = -H , \end{aligned}$$

The kinematic condition at the interface between the ice cover and the fluid

19 $$\begin{aligned} \eta _t - \phi _z = 0 \quad \text{ for } \quad x,y \in {\mathbb {R}}, \quad z = 0 , \end{aligned}$$

and the Bernoulli equation

20 $$\begin{aligned} \phi _t + g \eta + \frac{p}{\rho } = 0 \quad \text{ for } \quad x,y \in {\mathbb {R}}, \quad z = 0 . \end{aligned}$$

The pressure *p* is obtained from the beam equation for elastic solids. This equation is written as

$$\begin{aligned} {\mathscr {D}} \triangle_H^2 \eta - \frac{\rho _I h^3}{12} \triangle_H \partial_t^2 \eta + \rho _I h \partial _t^2 \eta + \rho _I g h + P - p = 0. \end{aligned}$$

Combining the last two equations yields the overall boundary condition as the linear version of (1):

21 $$\begin{aligned} \kappa g \triangle_H^{2} \eta - \frac{\rho _I h^3}{12 \rho } \partial _t^2 \triangle_H \eta + \frac{\rho _I h}{\rho } \partial _t^2 \eta + \frac{b}{\rho } \partial _t \eta + g \eta + \phi _t + \frac{P}{\rho } = 0. \end{aligned}$$

The resulting system is the hydro-elastic system in terms of two unknowns: the velocity potential $\phi$ in the fluid domain, and $\eta (x,y,t)$ which denotes the deflection of the ice cover from the rest position. As mentioned in Section “Mathematical model for hydro-elastic waves”, we denote the trace of the the potential by $\Phi (x,y,t) = \phi (x,y,0,t)$. The function $\Phi$ can be interpreted as Dirichlet boundary data for the Laplace equation on the fluid domain. On the other hand Neumann data for the Laplace equation are given by $\phi _z(x,y,0,t)$, and following^23^, these two function can be related by the so-called Dirichlet-to-Neumann operator $G_0$. As shown in^23^, this operator has the form (13). Using this operator, it can be shown directly that the equations can be reduced to the unknown $\Phi$ and $\eta$. The kinematic condition reduces to (2), and the visco-elastic Bernoulli equation can be written in terms of the operators $G_0$ and *K* as (3).

### The dispersion relation

It is useful to look at the propagation of harmonic plane waves in the system (17)–(18)–(19)–(21) without forcing and damping, i.e. in the case of $P=0$ and $b=0$. For plane waves, it is convenient to assume propagation in the direction of the *x*-axis, so that the free surface is of the form $\eta (x,t)= a \cos (kx - \omega t)$, where *a* is the amplitude, $k = 2\pi / \lambda$ is the wave number, $\lambda$ is the wavelength, $\omega = 2 \pi / T$ is the radial frequency, and *T* is the period. The corresponding velocity potential is given by $\phi = \frac{a \omega }{k} \frac{ \cosh (k (z+H))}{\sinh (H)} \sin (kx - \omega t)$. Substitution into the dynamic boundary condition (1) with $b=0$ and $P=0$ yields the dispersion relation

22 $$\begin{aligned} c^2(k) = \frac{g/k + {\mathscr {D}} k^3 / \rho }{\coth {k H} + h k \rho _{I}/\rho } \end{aligned}$$

In terms of the phase speed $c = \omega /k$ (Fig. 7).
